# Thiamine diphosphate adenylyl transferase from *E. coli*: functional characterization of the enzyme synthesizing adenosine thiamine triphosphate

**DOI:** 10.1186/1471-2091-8-17

**Published:** 2007-08-16

**Authors:** Alexander F Makarchikov, Alain Brans, Lucien Bettendorff

**Affiliations:** 1 Center for Cellular and Molecular Neurobiology, University of Liège, Avenue de l'Hôpital 1, B-4000 Liège, Belgium; 2 Chemical Department, Grodno State Agricultural University, Tereshkova St.28, Grodno 230008, Belarus; 3 Center for Protein Engineering, University of Liège, Allée de la Chimie 6, B-4000 Liège, Belgium

## Abstract

**Background:**

We have recently identified a new thiamine derivative, adenosine thiamine triphosphate (AThTP), in *E. coli*. In intact bacteria, this nucleotide is synthesized only in the absence of a metabolizable carbon source and quickly disappears as soon as the cells receive a carbon source such as glucose. Thus, we hypothesized that AThTP may be a signal produced in response to carbon starvation.

**Results:**

Here we show that, in bacterial extracts, the biosynthesis of AThTP is carried out from thiamine diphosphate (ThDP) and ADP or ATP by a soluble high molecular mass nucleotidyl transferase. We partially purified this enzyme and characterized some of its functional properties. The enzyme activity had an absolute requirement for divalent metal ions, such as Mn^2+ ^or Mg^2+^, as well as for a heat-stable soluble activator present in bacterial extracts. The enzyme has a pH optimum of 6.5–7.0 and a high *K*_m _for ThDP (5 mM), suggesting that, *in vivo*, the rate of AThTP synthesis is proportional to the free ThDP concentration. When ADP was used as the variable substrate at a fixed ThDP concentration, a sigmoid curve was obtained, with a Hill coefficient of 2.1 and an *S*_0.5 _value of 0.08 mM. The specificity of the AThTP synthesizing enzyme with respect to nucleotide substrate is restricted to ATP/ADP, and only ThDP can serve as the second substrate of the reaction. We tentatively named this enzyme ThDP adenylyl transferase (EC 2.7.7.65).

**Conclusion:**

This is the first demonstration of an enzyme activity transferring a nucleotidyl group on thiamine diphosphate to produce AThTP. The existence of a mechanism for the enzymatic synthesis of this compound is in agreement with the hypothesis of a non-cofactor role for thiamine derivatives in living cells.

## Background

Thiamine and its phosphorylated derivatives are common cellular constituents in all living forms studied so far [1]. While the role of thiamine diphosphate (ThDP) as a cofactor for more than 25 enzymes is well documented [2], we have so far little information concerning the possible role(s) of other thiamine derivatives.

No known biological role has been documented for thiamine monophosphate (ThMP), but recent results suggest a role for thiamine triphosphate (ThTP). Although it is only a minor component (0.1 – 1 %) of total thiamine in most tissues, ThTP was found in all organisms investigated so far [1]. In plants and in bacteria, the appearance of ThTP seems to be a response to specific conditions of cellular stress [1,3]. In *E. coli *for instance, the initial accumulation of ThTP appears to be required for optimal growth in media containing a carbon source but no amino acids.

Recently, we identified a new thiamine derivative, adenosine thiamine triphosphate (AThTP). This compound was first discovered in *E. coli*, but it is also present in low amounts in plants and animals [4]. Like ThTP, AThTP appears to be a signal produced in bacteria in response to some form of cellular stress; however, the two compounds are formed under different conditions and generally do not accumulate simultaneously. Both are hardly detectable when the bacteria are grown in rich media under optimal conditions. When the bacteria are transferred to minimal M9 medium, AThTP appears in the absence of any carbon source and it quickly disappears when glucose is added, suggesting that it is produced in response to carbon starvation. In contrast, ThTP synthesis requires the presence of an energy substrate such as glucose.

Although the presence of ThTP in many tissues has been known for over 50 years, the mechanism of its enzymatic synthesis remains unclear. In particular, no significant net synthesis of ThTP could be detected so far using cell-free extracts of *E. coli*. In contrast, we observed a synthesis of AThTP from ADP and ThDP in soluble fractions from sonicated bacteria. Here, we describe the partial purification and some kinetic properties of a high molecular weight enzyme (or enzyme complex) catalyzing the synthesis of AThTP in *E. coli*.

## Results

### Partial purification of AThTP-synthesizing enzyme from E. coli

*E. coli *(strain BL21) were grown aerobically overnight in LB medium either in a 15 l fermentor (BioFlo 4500, New Brunswick Scientific Company, Edison, NJ, USA) under constant aeration (1 VVM, 37°C) and agitation (400 rpm) or in 1 liter flasks (37°C, 250 rpm). The cells were sedimented (10 min, 10 000 × g), suspended in 500 ml of minimal M9 medium containing 10 mM glucose and incubated for 40 min (37°C, 250 rpm). Bacteria were collected by centrifugation (10 min, 10 000 × g), suspended in 30 ml of 50 mM Tris-HCl buffer, pH 7.4, containing 0.2 mM EDTA, 0.1 mM phenylmethylsulfonyl fluoride, 0.15 M KCl, and frozen at -20°C. After thawing the suspension was sonicated (100 kHz, 3 × 60 sec, on ice), the pellet was removed by centrifugation (30 min, 15 000 × g), and the supernatant was used as a source for enzyme purification.

**Figure 1:**
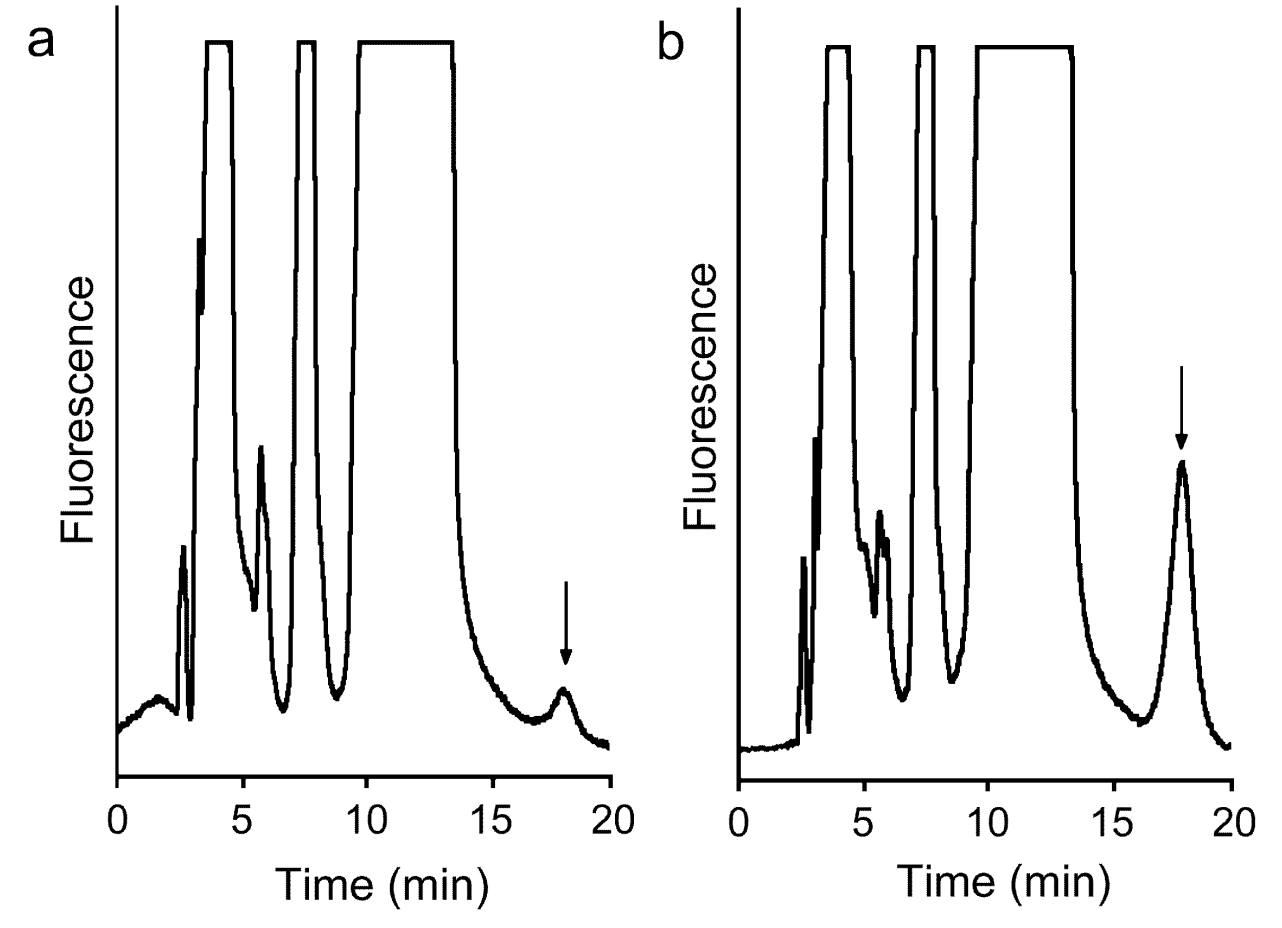
**Chromatograms showing the synthesis of AThTP (arrow) in the high molecular mass fraction (Sephadex G-200, peak I)**. The incubation was carried out for 1 h under the conditions described in the Methods secion in the absence (a) and the presence (b) of 1 mM ADP. The flow rate was 0.5 ml/min.

The extract was placed in a water bath (55°C) under continuous stirring and heated to 50°C. After 5 min, the sample was placed on ice, cooled to 4°C and the precipitate was removed by centrifugation (10 min, 15 000 × g). The supernatant was concentrated to 5.0 ml with Centriplus 10 centrifugal filter units (Amicon Inc., Beverly, MA, USA) and run on a Sephadex G-200 column (∅ 2.4 × 65 cm) calibrated with protein size standards. The chromatography was carried out in 20 mM Tris-HCl buffer, pH 7.4, containing 0.2 mM EDTA and 0.1 M NaCl, at a flow rate of 5 cm . hr^-1^, and 4-ml fractions were collected. AThTP-synthesizing activity was eluted in two nearly equal peaks, corresponding to molecular masses of 355 ± 14 kDa and 190 ± 4 kDa (*n *= 2). The first peak was used for subsequent kinetic studies. The purification data are summarized in Table 1. Fig. 1 shows the synthesis of AThTP in the high molecular mass fraction.

**Table 1:** Partial purification of AThTP-synthesizing enzyme from *E. coli*

Fraction	Volume (ml)	Total protein (mg)	Total activity (pmol.min^-1^)	Specific activity (pmol.min^-1^.mg^-1^)	Purification factor
Extract	32.0	99.2	436.5	4.4	1
Heat treatment	31.0	71.3	370.8	5.2	1.2
Sephadex G-200:					
Peak I (maximum)	4.0	0.68	63.4	93.2	21.2
Peak II (maximum)	4.0	0.94	75.4	80.2	18.2

The purification factor was calculated by comparing the activity in a given fraction to the activity found in the extract.

### Kinetic properties

**Figure 2:**
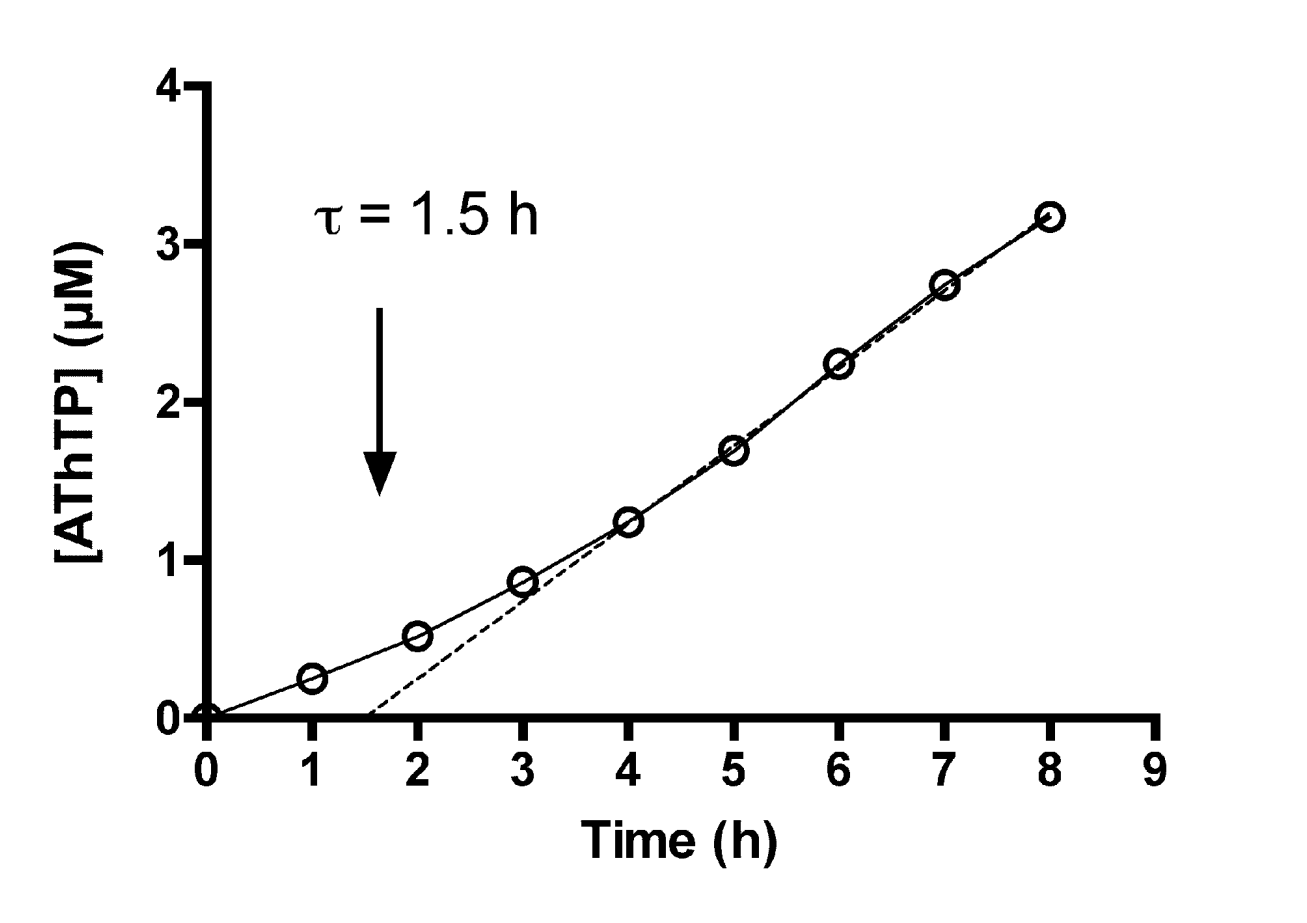
**Time dependence of AThTP synthesis. The experiment was carried out under standard **conditions (1 mM ADP, 0.1 mM ThDP, 10 mM Mg^2+^, maleate buffer, pH 6.5), and the protein concentration was 100 μg/ml.

As shown in Fig. 2, under standard conditions, AThTP synthesis was not linear with time. Instead, we observed a pronounced lag period in the accumulation of product, allowing no initial rate measurements to be made. The duration of the lag period, τ, as determined by extrapolation of the linear part of the curve to the time axis, was about 1.5 hour.

**Figure 3:**
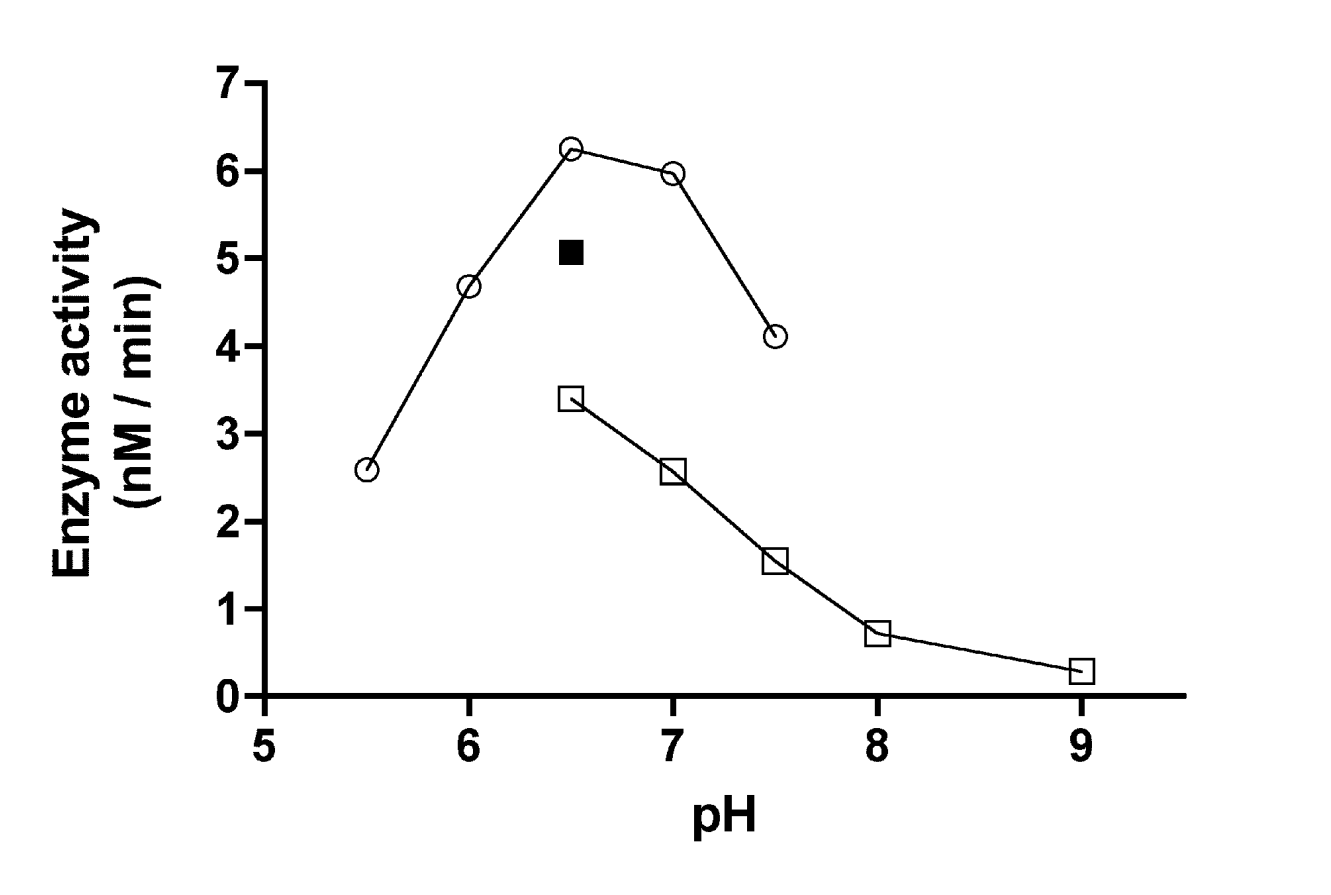
**Effect of pH on AThTP synthesizing enzyme**. The Mg^2+ ^concentration was 10 mM, and the buffers used were as follows: (○) 50 mM acetate, pH 5.5; 50 mM maleate, pH 6.0–6.5; 50 mM Tris-maleate, pH 7.0; 50 mM Tris-HCl, pH 7.5; (■) 50 mM Bis-Tris-propane; (□) 100 mM Bis-Tris-propane.

The influence of hydrogen ion concentration on the enzyme activity was examined at pH values ranging from 5.5 to 9.0. Acetate (50 mM, pH 5.5), maleate (50 mM, pH 6.0–6.5), Tris-maleate (50 mM, pH 7.0), Tris-HCl (50 mM, pH 7.5) and Bis-Tris-propane (50 mM, pH 6.5; 100 mM, pH 6.5–9.0) buffers were used in the assay mixture. As can be seen in Fig. 3, the enzyme has a pH optimum of 6.5 to 7.0 and there is clearly an inhibitory effect of Bis-Tris-propane on the enzyme activity.

**Figure 4:**
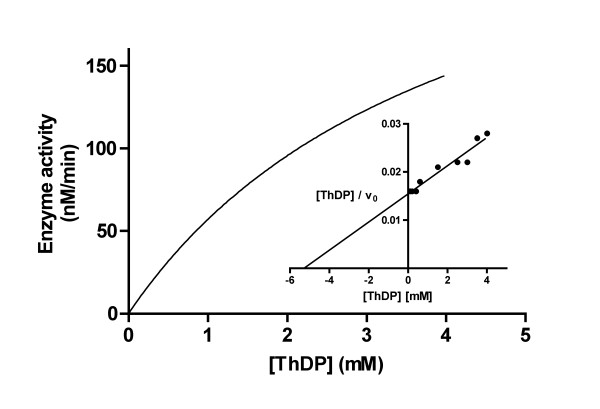
**Effect of ThDP concentration on the activity of AThTP-synthesizing enzyme at fixed concentrations of ADP (1 mM) and Mg**^2+^**(10 mM). **The continuous line was obtained by nonlinear regression using the Michaelis-Menten equation with an apparent *K*_m _of 4.2 ± 0.5 mM. Inset shows the Hanes plot of the data and the line was obtained by linear regression. The results are expressed as mean ± SD for 3 experiments.

The effect of ThDP concentration on the reaction rate was studied within the range of 0.1 to 4 mM at a fixed ADP concentration of 1 mM. The reaction followed Michaelis-Menten kinetics giving a hyperbolic saturation curve (Fig. 4) with an apparent *K*_m _value of 4.2 ± 0.5 mM (n = 3) obtained from the direct plot using non-linear regression. Extrapolation from the Hanes plot gave a value of 5.2 mM (Fig. 4, inset).

**Figure 5:**
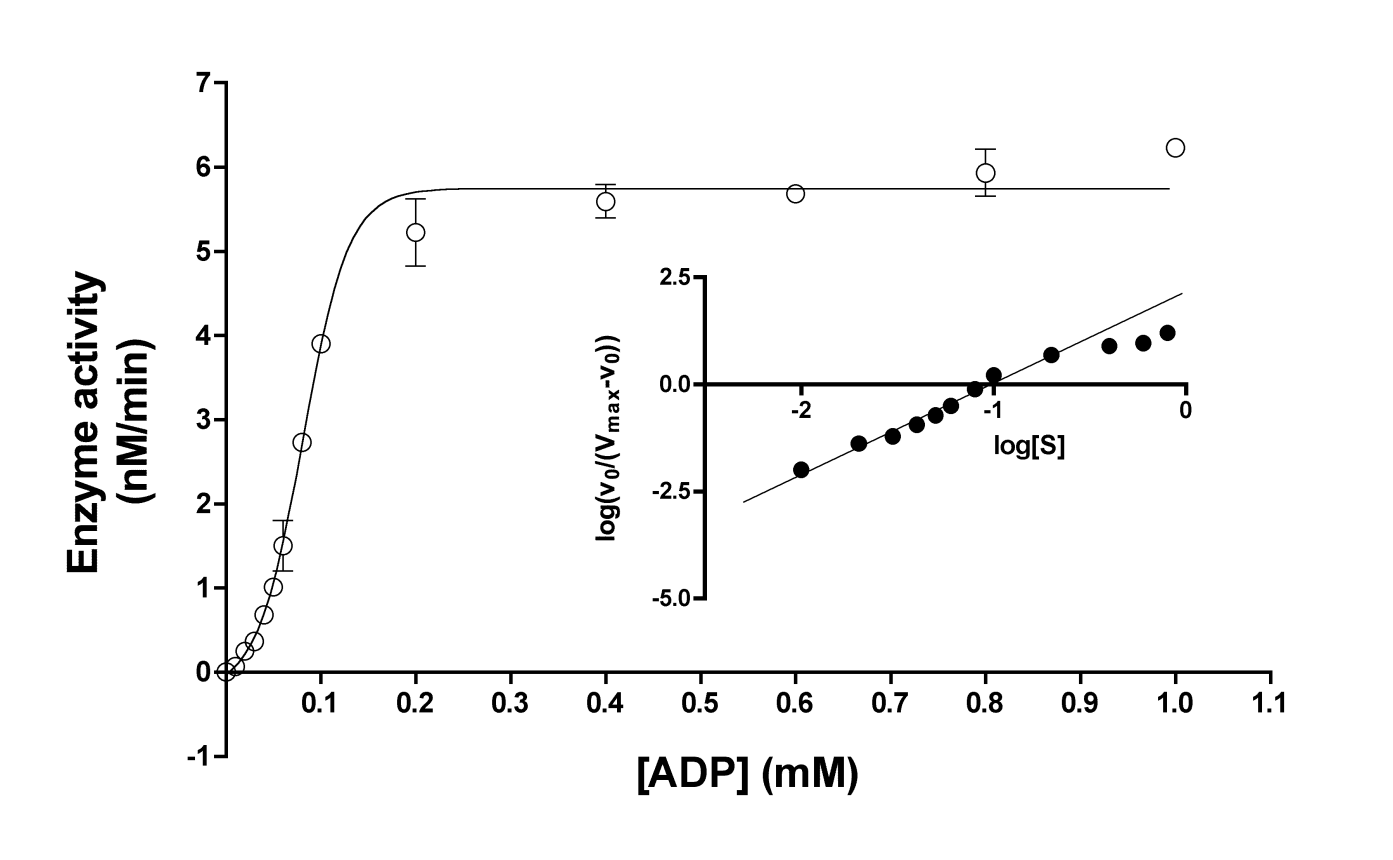
**Effect of varying ADP concentrations on the activity of AThTP-synthesizing enzyme at a fixed ThDP concentration of 0.1 mM and 10 mM Mg^2+^**. The results are the mean ± SD for 3 to 6 experiments. The line was obtained by nonlinear regression assuming a sigmoid dose-response curve with an *EC*_50 _of 0.08 mM. The inset shows a Hill plot obtained for ADP concentrations ranging from 0.04 to 0.6 mM. The Hill coefficient (n_H _= 2.1) was calculated from the slope of the regression line over the linear portion of the graph (0.01 – 0.20 mM ADP).

Fig. 5 illustrates the effect of increasing ADP concentrations on the rate of AThTP synthesis at a fixed concentration of ThDP (0.1 mM). In contrast to the effect of ThDP concentration, varying the ADP concentration did not yield Michealis-Menten kinetics but a sigmoid curve was observed. An S_0.5 _value of 0.08 mM was estimated, indicating that the apparent affinity of the active sites for ADP is rather high. The n_H _coefficient calculated from the Hill plot (Fig. 5, inset) was 2.1, in agreement with the possibility of two cooperative binding sites for ADP.

**Figure 6:**
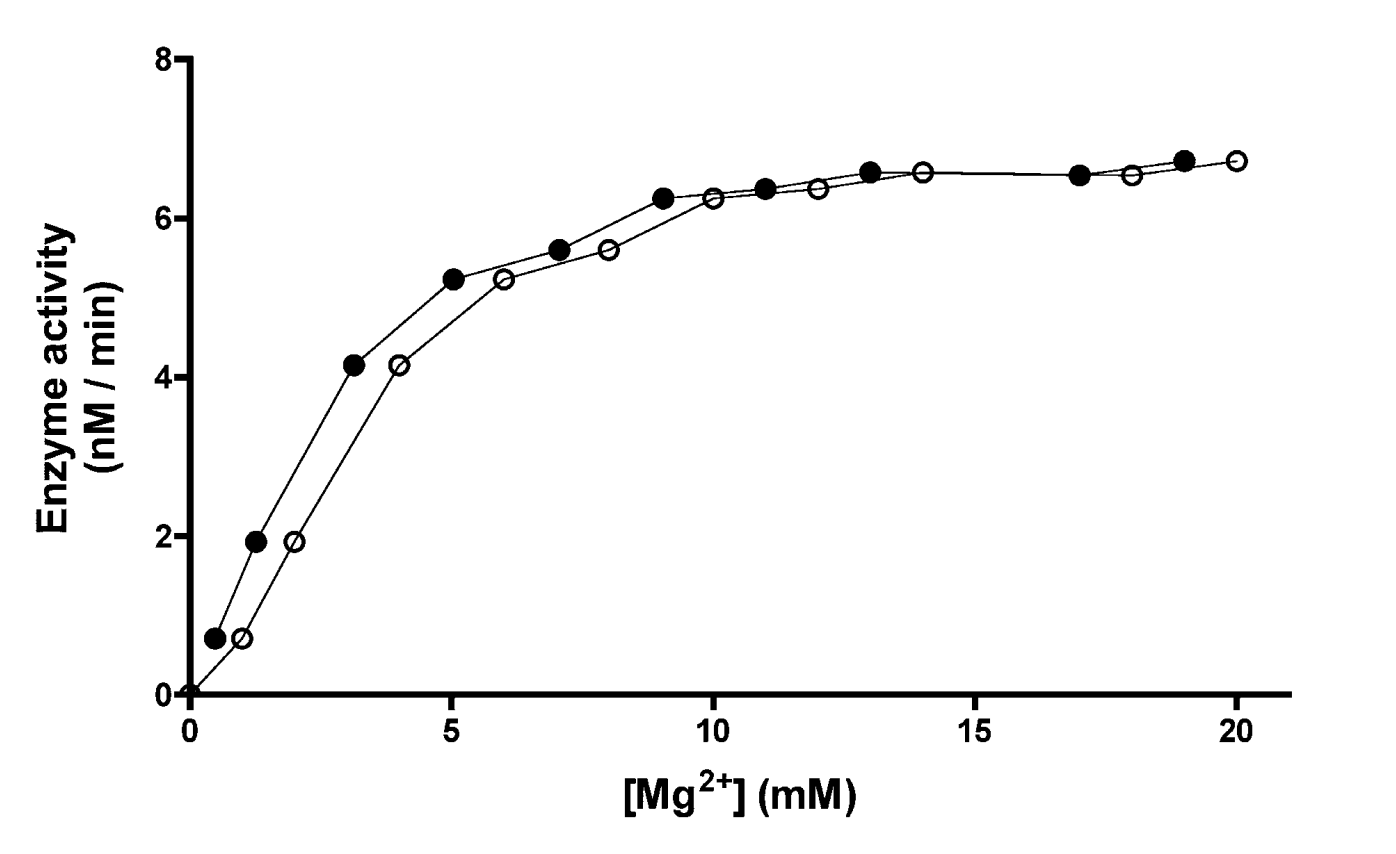
**Effect of Mg^2+ ^concentration on the activity of AThTP-synthesizing enzyme at a fixed ThDP concentration of 0.1 mM and 1 mM ADP**. Total Mg^2+ ^concentration (○); free Mg^2+ ^concentration (●).

No measurable AThTP synthesis was observed in the absence of divalent metal ions. Among the cations tested (Ca^2+^, Mg^2+ ^or Mn^2+^, each at 5 mM), Mn^2+ ^was the most efficient; Mg^2+ ^was 70 % less effective, whereas no measurable AThTP synthesis was observed in the presence of Ca^2+^. The effect of varying Mg^2+ ^concentrations on the reaction rate was explored at a fixed ThDP concentration of 0.1 mM and ADP concentration of 1 mM. As shown in Fig. 6, the saturation curve was sigmoid when total Mg^2+ ^concentrations were used. This sigmoidicity did not disappear completely when free Mg^2+ ^concentrations (calculated assuming a dissociation constant of 457 μM for Mg- ADP- complex [5], were used for analysis. The apparent values of *S*_0.5 _for total and free Mg^2+ ^were estimated to be about 2.8 mM and 2.0 mM, respectively.

### Substrate specificity

Various combinations of substrates were tested: ADP + ThMP, ADP + ThDP, ADP + ThTP, ATP + ThMP, ATP + ThDP, ATP + ThTP. Among the thiamine phosphates, the enzyme exhibited an absolute specificity for ThDP, whereas both ADP and ATP could serve as the second substrate, the rate of AThTP synthesis being essentially equal with either substrate (data not shown). Our preparation contained no significant ATP hydrolyzing activity, excluding that the activity observed in the presence of ATP was due to its hydrolysis to ADP. No peaks corresponding to a newly synthesized compound were observed on chromatograms when ADP was replaced by GDP, CDP or UDP. There was also no synthesis of compounds such as diadenosine phosphates when a single substrate such as ADP or ATP was used (in this case the reaction was monitored by UV detection after separation on a C18-reversed-phase HPLC column). ThDP alone did not appear to be transformed either.

## Discussion

AThTP has recently been discovered in *E. coli*, where it accumulates as a result of carbon starvation [4]. Here, we describe for the first time the existence, the partial purification and the kinetic properties of an AThTP-synthesizing enzyme. The activity was low in *E. coli *extracts (supernatant obtained after sonication and centrifugation): 4.1 pmol·min^-1^·mg^-1 ^of protein under standard incubation conditions. We tried to concentrate the enzyme, by using different precipitation procedures, including ammonium sulfate, acetone, polyethylene glycol and isoelectric point precipitation, but all of them were unsuccessful; each time a 5–10-fold reduction in enzyme activity was observed. We considered that this loss of activity could be due either to inactivation of the enzyme or to the removal of an essential activator. It seems that both factors are important. We found that addition of a boiled bacterial extract to the incubation medium containing salted-out enzyme led to 40–50% increase in the reaction rate (data not shown), suggesting the requirement for an activator, whose chemical nature remains unknown, though it seems to be a low molecular weight compound resistant to heating. Recent studies show an important accumulation of cAMP and phosphoenolpyruvate during carbon starvation [6], but none had an activating effect on AThTP synthesis (not shown).

Nevertheless, even in the presence of the activator, the specific activity of the enzyme preparation remained low. We faced the same problem testing conventional adsorption chromatography methods and resins – Phenyl-Sepharose, DEAE-Sephacel, Blue-Sepharose, 2',5'ADP-Sepharose and hydroxyapatite. Each time, the activity was lost after chromatography.

It should be noted that this loss of activity was not the consequence of proteolysis or unfavorable buffer composition, as the extract could be kept at +4°C for several days without any significant loss of activity. Moreover, the use of common protective reagents such as glycerol or dithiothreitol did not lead to increased enzyme recovery. It seems that the AThTP-synthesizing enzyme is extremely sensitive to any separation by procedures based on precipitation or adsorption. On the other hand, this enzyme was rather resistant to heating and liquid-liquid chromatography such as gel filtration. Therefore, we set up a procedure for the partial purification in two steps, heat treatment and gel filtration on Sephadex-G-200.

During the gel-filtration step, two peaks of activity were eluted from the column, with molecular masses of 355 and 190 kDa respectively. It should be noted that the respective peak areas depended on the experimental conditions, especially on the time of sample processing and protein concentration, the high molecular mass peak being predominant in most cases. One may suppose that these peaks correspond to aggregated and dissociated forms of the enzyme, based on the ratio of their molecular masses. The effect of ADP concentration (Fig. 5) gave a sigmoid curve with a Hill coefficient around 2, which is in agreement with the assumption that the 355-kDa enzyme complex contains two cooperative subunits.

The AThTP-synthesizing enzyme also appears to undergo time-dependent reorganization. As shown in Fig. 2, it took several hours before the activity reached a steady-state, pointing to slow conformational changes in the molecular structure of the enzyme. Such a behavior is characteristic of a special class of allosteric enzymes capable of slow association-dissociation processes induced by substrate, ligands or protein concentration [7]. It is possible that further dissociation of the 190 kDa species into smaller subunits is responsible for the low stability of the AThTP-synthesizing enzyme during precipitation and adsorption procedures, indicating a weak binding between its subunits.

The partially purified enzyme exhibited a maximal activity at pH 6.5–7.0 (Fig. 3), the enzyme being active in a rather broad range of pH. The enzyme is sensitive to the nature of the buffer as well as the buffer concentration. At pH 6.5, for example, the activity in 50 mM maleate was 1.2 times higher than in 50 mM Bis-Tris-propane, and the latter, in turn, was 1.5 times higher than in the 100 mM Bis-Tris-propane.

The enzyme showed hyperbolic saturation with respect to ThDP concentration at a fixed ADP concentration, with an apparent *K*_m _of approximately 5 mM (Fig. 4). Though this is a very high value compared to the free intracellular ThDP concentration in bacteria [1], it has a physiological meaning. Indeed, under such conditions ([*S*] <<*K*_m_) the reaction follows first order kinetics with respect to ThDP: the more ThDP is available, the more AThTP can be synthesized. This could explain why in intact bacteria, AThTP accumulates during carbon starvation [4]. In this situation, where catabolic processes become prevalent, cellular proteins are degraded, probably leading to the dissociation of enzyme-bound ThDP and a substantial increase in the cytosolic concentration of free ThDP.

On the other hand, a sigmoid saturation curve, a typical feature of allosteric enzymes, was obtained for ADP at a fixed ThDP concentration (Fig. 5). This might indicate a regulatory mechanism. However, ADP concentration in bacterial cells is around 1 mM [8] indicating that the enzyme is saturated under normal physiological conditions.

The enzyme has an absolute dependence on divalent metal ions such as Mg^2+^or Mn^2+^. A sigmoid saturation curve was observed when the rate of AThTP synthesis was plotted against the total Mg^2+ ^concentration (Fig. 6). As Mg^2+ ^is known to form complexes with polyphosphates, the concentration of free Mg^2+ ^is not equal to its total concentration and this could explain the reason for the sigmoid behavior. Indeed, if we replace total Mg^2+ ^by free Mg^2+ ^concentration estimated from the dissociation constant of 457 μM [5] for the Mg- ADP^- ^complex, the shape of the plot becomes less sigmoid (Fig. 6). In addition, complexes with ThDP (*K*_d _= 420 μM [9]) and buffer ions (maleate) are also generated, leading to a further decrease in free Mg^2+ ^content, especially in the range of its low concentrations. It is thus likely that there are no cooperative effects of Mg^2+ ^ions.

This enzyme is highly specific for ThDP among thiamine phosphates, but it is able to use both ATP and ADP as the second substrate. As AThTP is synthesized only under conditions of carbon starvation [4], i.e. when ATP content is low, ADP is probably the physiologically relevant substrate. The replacement of ADP (ATP) by GDP, UDP or CDP gave no product formation. It could be argued that, as the apparent affinity for ThDP is low, ThDP might not be the physiological substrate. An obvious possibility is that the real substrate is a second ADP molecule instead of ThDP. An analogous phenomenon has been reported in the case of adenylate kinase 1: ThDP can replace ADP at one site but is a much poorer substrate [10]. If this were the case for our enzyme an important synthesis of diadenosine triphosphate should be observed with ADP as the sole substrate, but this was not the case. Moreover, the synthesis of AThTP should be impaired when ADP is in excess over ThDP. However, data in Fig. 5 show that there is no tendency to inhibition of AThTP synthesis by excess ADP. Those data strongly suggest that ADP does not bind to the ThDP-binding site with high affinity. This does not exclude, however, that some unknown substrate might replace ThDP.

Concerning the correct systematic name of AThTP-synthesizing enzyme, it should belong to EC subgroup of 2.7.7 of nucleotidyl transferases as a nucleotidyl moiety is transferred to ThDP. Subgroup 2.7.7 comprises a set of enzymes carrying out a nucleotidyl transfer and release of inorganic phosphate (with an NDP as substrate) or pyrophosphate (with an NTP as substrate): X- P- P(- P) + P- Y ⇔ X- P- P- Y + P(- P). Correspondingly, the AThTP-synthesizing enzyme could be named ADP (ATP): thiamine diphosphate adenylyl transferase (EC 2.7.7.65). As both ADP and ATP can act as substrates, we recommend the name of ThDP adenylyl transferase (THAT).

## Conclusion

The discovery of a new thiamine compound, AThTP [4], as well as of its synthesizing enzyme in the present study, along with the recent findings concerning ThTP [1,3,11] and the discovery of riboswitches [12,13] underline the great diversity of thiamine biochemistry and is strongly in favor of one or several non-cofactor roles of thiamine derivatives in living organisms.

## Methods

### Chemicals

Sephadex G-200 was supplied by Amersham Biosciences (Little Chalfont, UK). Diethyl ether and all other solvents (HPLC grade) were from Biosolve (Valkenswaard, The Netherlands). All other reagents were from Sigma-Aldrich (Bornem, Belgium) or Merck KGaA (Darmstadt, Germany). All solutions were prepared with milli-Q water (Millipore S.A./N.V., Brussels, Belgium).

### Activator preparation

E. coli (strain BL21) were grown overnight (37°C, 250 rpm) in Luria-Bertani (LB) medium (tryptone, 10 g/l; yeast extract, 5 g/l; NaCl, 10 g/l, pH 7.0), collected by centrifugation (10 min, 10 000 × g), and suspended in half the initial volume of M9 minimum medium (Na_2_HPO_4_, 6 g/l; KH_2_PO_4_, 3 g/l; NaCl, 0.5 g/l; NH_4_Cl, 1 g/l; CaCl_2_, 3 mg/l; MgSO_4_, 1 mM, pH 7.0) containing 10 mM glucose. The culture was incubated for 40 min (37°C, 250 rpm) and centrifuged (10 min, 10 000 × g). The pellet was suspended in 50 mM Tris-HCl buffer (1/33 of the volume of M9 culture), pH 7.4, containing 0.2 mM EDTA, 0.1 M KCl, and frozen at -20°C. Then the cells were thawed, disrupted by sonication (100 kHz, 3 × 1 min) on ice and centrifuged for 30 min at 15 000 × g. The supernatant was boiled for 3 min, put on ice, the precipitate was sedimented (5 min, 15 000 × g), and the final supernatant was used as an activator for enzyme assays. Protein concentrations were measured by the method of Bradford [14] or from the absorbance at 280 nm.

### Determination of enzyme activity

The standard incubation mixture for AThTP synthesis contained 50 mM sodium maleate, pH 6.5, 1 mM ADP, 0.1 mM ThDP, 10 mM MgSO_4_, and aliquots of enzyme preparation and 10 μl activator in a final volume of 0.1 ml. Any changes in the protocol are indicated in the legends to the figures. The reaction was carried out at 37°C for 1 h and stopped by addition of 0.5 ml of 12% TCA followed by extraction of the acid with 3 × 1.5 ml of diethyl ether. AThTP was quantified using a HPLC method as previously described [4,15]. Briefly, a 40-μl aliquot of sample was oxidized with 25 μl of 4.3 mM potassium ferricyanide in 15% NaOH and injected into the HPLC system (System 522, Kontron Instruments, Milan, Italy) equipped with a PRP-1 column (∅ 4.1 × 150 mm, Hamilton Co., Reno, NV, USA) protected by a guard column (Hamilton) and a SFM 25 spectrofluorimeter (Kontron Instruments). The separation was performed at a flow rate of 0.5 ml . min^-1 ^in a mobile phase containing 50 mM NaH_2_PO_4_, pH 9.5, 25 mM tetra-*n*-butylammonium hydrogen sulfate and 4% tetrahydrofuran. The peak areas were calculated using the KromaSystem 2000 software (Bio-Tek Kontron Instruments) and compared to the area of a standard solution of chemically synthesized AThTP [4].

Adenine nucleotides were monitored by UV detection (254 nm) after separation on a 5 μm Chromspher C18 column (150 × 4.6 mm, Varian D.V., Middelburg, The Netherlands). The mobile phase was composed of 25 mM tetra-*n*-butylammonium hydrogen sulfate, 50 mM NaH_2_PO_4 _adjusted to pH 7.0 and 15 % methanol. The flow rate was 1 ml/min.

Data analysis was performed using GraphPad Prism version 4.00 for Macintosh, GraphPad Software, San Diego California USA.

### Determination of molecular mass on a Sephadex G-200 column

The proteins were separated at 4°C on a Sephadex G-200 column (∅ 2.4 × 65 cm) equilibrated with 20 mM Tris-HCl, pH 7.4, containing 0.1 M NaCl, at a flow rate of 5 cm . hr^-1^. The column was calibrated with the following standard proteins: apoferritin (443 kDa), *β*-amylase (200 kDa), alcohol dehydrogenase (150 kDa), bovine serum albumin (66 kDa) and carbonic anhydrase (29 kDa). The elution volume (*V*_e_) of AThTP-synthesizing enzyme was estimated from its activity and its molecular mass was calculated from the plot of log*M*_r _versus log*V*_e_/*V*_0_.

## Abbreviations

AThTP adenosine thiamine triphosphate

ThMP thiamine monophosphate

ThDP thiamine diphosphate

ThTP thiamine triphosphate

## Authors' contributions

AFM made most of the experimental work described in the study and wrote the first draft of the manuscript. AB was responsible for the large-scale production of *E. coli *using fermentators. LB was the project leader, coordinated the study, participated in its design and wrote the final manuscript. All authors read and approved the final manuscript.
